# Multi-Responsive SEBS/MXene Janus Membranes Enabling Piezoelectric Energy Harvesting, Humidity Sensing, and Infrared Stealth

**DOI:** 10.1007/s40820-026-02184-x

**Published:** 2026-05-11

**Authors:** Weiwen Wang, Hong Ma, Lun Zhang, Jihai Zhang, Aimin Zhang

**Affiliations:** 1 State Key Laboratory of Advanced Polymer Materials, Polymer Research Institute, Sichuan University, Chengdu, 610065 People’s Republic of China; 2 Hubei Three Gorges Laboratory, Yichang, 443007 Hubei People’s Republic of China

**Keywords:** Janus membrane, Energy harvesting, Humidity sensing, Infrared stealth, Multi-functional integration

## Abstract

In response to the urgent need for multifunctional integration in flexible intelligent systems, as well as the limitations of traditional multilayer integration strategies in terms of interface and mechanical performance, inspired by the intrinsic asymmetry of natural Janus structures, this study designed and prepared a novel flexible styrene–ethylene–butylene–styrene (SEBS)/MXene (SM) Janus fibrous membrane. Under mechanical pressure, the SM Janus membrane can generate a remarkable piezoelectric output of up to 28 V and 133 nA, sufficient to drive humidity sensing, thereby establishing a self-powered sensing-energy supply closed-loop system. The synergistic interaction between the hydrophilic MXene layer and the hydrophobic SEBS layer enables the humidity sensor to exhibit high sensitivity and rapid response characteristics, with response and recovery times of 0.79 and 0.35 s, respectively. This allows for effective monitoring of respiration, ambient humidity, and speech signals. Furthermore, the membrane demonstrates exceptional infrared camouflage performance, with an emissivity as low as 32%, significantly modulating the apparent temperature of targets. This study pioneers the integration of energy harvesting, self-powered sensing, and adaptive camouflage into a single material platform, showcasing broad application prospects in fields such as bionic electronic skin and environmental interactive systems.

**Supplementary Information:**

The online version contains supplementary material available at 10.1007/s40820-026-02184-x.

## Highlights

- Styrene–ethylene–butylene–styrene copolymer/MXene Janus membranes prepared via electrospinning–vacuum filtration enable synergistic integration of energy harvesting, self-powered sensing, and stealth technology.
- Piezoelectric output (28 V/133 nA) drives self-powered humidity sensing with response recovery as fast as 0.79/0.35 s.
- Low infrared emissivity achieves precise regulation of target apparent temperature, demonstrating outstanding stealth performance.

**Supplementary Information:**

The online version contains supplementary material available at 10.1007/s40820-026-02184-x.

## Graphical abstract

**Figure:**
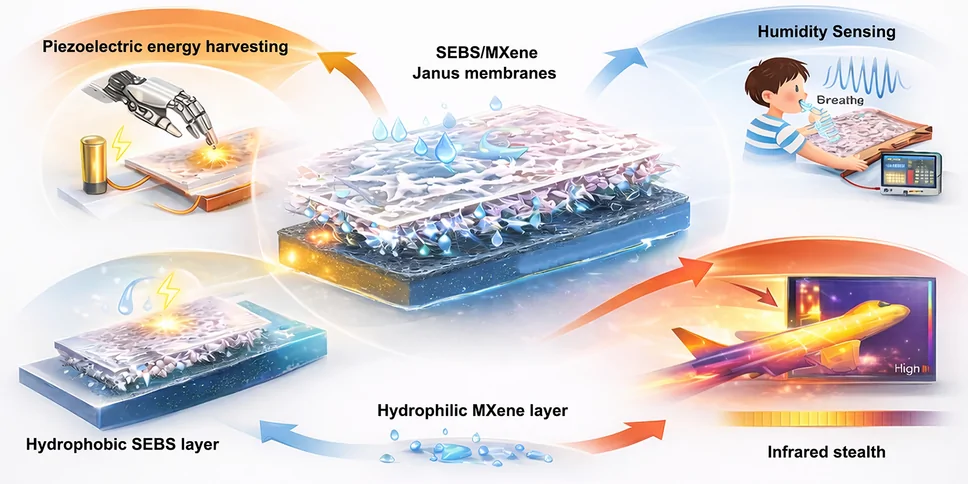

## Introduction

The rapid advancement of flexible electronics, smart wearable devices, and adaptive camouflage technologies has spurred growing interest in the development of new-generation multifunctional integrated systems [1–5]. Such systems are capable of synergistically sensing and intelligently responding to diverse external stimuli such as pressure, humidity, and light/heat [6–10]. However, conventional single-function devices, often designed for specific applications, struggle to meet the demands of complex multitask environments [11–13]. Moreover, traditional integration strategies typically rely on multilayer stacking or heterogeneous structural bonding, which inevitably introduce bottlenecks such as poor interfacial compatibility, mechanical mismatch, and complex manufacturing processes [14–17]. Therefore, a key challenge in the field remains how to achieve the collaborative integration of multiple functions, such as energy management, sensing, and environmental interaction, within the same material platform via a simple and efficient structural design.

Inspired by the intrinsic asymmetry of natural Janus structures, artificial Janus membranes offer a revolutionary design concept for enabling directional transport, logic-based responses, and functional integration within a single device, owing to their inherent physicochemical heterogeneity on opposite sides [18–21]. By precisely controlling the composition and microstructure of each side, the two surfaces can independently respond to different stimuli or perform distinct tasks, thereby facilitating complex sense-decide-act cycles within a monolithic membrane [22–25]. Nonetheless, identifying compatible and functionally complementary building units, along with developing scalable fabrication strategies to achieve structurally precise Janus membranes, remains a central focus of current research.

Compared to other thermoplastic elastomers (TPEs), styrene–ethylene–butylene–styrene copolymer (SEBS) stands out with unique advantages that make it an ideal electrospinning substrate for the multifunctional SEBS/MXene Janus membrane. Based on the above considerations, this study ingeniously designed and prepared a novel flexible SEBS/MXene (SM) Janus fiber membrane. This structure employs a highly elastic SEBS electrospun fiber membrane as the flexible substrate and supporting framework, while a continuous and dense MXene (Ti_3_C_2_T_x_) functional layer is precisely constructed on its surface via vacuum-assisted filtration technology. These advantages are closely tied to the material’s intrinsic properties and the collaborative requirements of piezoelectric energy harvesting, humidity sensing, and infrared stealth functionalities. The inherent hydrophobicity and elasticity of the SEBS layer [26–28] complement perfectly the excellent hydrophilicity, high electrical conductivity, and low emissivity of the MXene layer [29–32], together laying a material foundation for integrating piezoelectric response, humidity sensing, and near-infrared (NIR) camouflage functionalities.

Specifically, under external mechanical pressure, the SM Janus membrane generates a piezoelectric response via interfacial charge separation and accumulation, enabling effective charging and discharging behaviors. This piezoelectric output simultaneously serves as the driving energy for the humidity sensing function, eliminating the reliance on an external power supply and enhancing the portability and practicality of the device. Benefiting from the functional synergy of the Janus structure, the hydrophilic MXene layer rapidly adsorbs environmental water molecules, which induces a significant change in electrical resistance, while the hydrophobic SEBS backsheet effectively blocks interference from external moisture, preventing unwanted signal distortion. The synergistic effect between these two components significantly improves the response speed, selectivity, and signal stability of the sensor, ultimately constructing a self-powered closed-loop system integrating mechanical energy harvesting, energy storage, and sensing power supply into a single platform. Furthermore, the MXene layer exhibits low emissivity in the mid-infrared region, significantly reducing the thermal signature of the target. In conclusion, this study provides an innovative design strategy for multifunctional integrated flexible smart platforms, which holds broad application prospects in fields such as artificial electronic skin, environmental monitoring, and adaptive camouflage systems.

## Experimental Section

### Materials

Thermoplastic elastomer styrene–ethylene–butylene–styrene copolymer (SEBS, YH-503 T) was synthesis from Sinopec Baling Petrochemical Co., Ltd. Tetrahydrofuran (THF) was obtained from Chengdu Changlian Chemical Reagent Co., Ltd. Lithium fluoride (LiF, 99%) was bought from Adamas Co., Ltd. Hydrochloric acid (HCl, 36.0–38.0 wt%) was supplied by Chengdu Cologne Chemical Co., Ltd. Ti_3_AlC_2_ powder (200 mesh) was purchased from Shanghai Titan Technology Co., Ltd. All chemicals were of analytical grade and were used as received without further modification. All aqueous solutions were prepared using Milli-Q-grade (> 18.2 mΩ cm^−1^) deionized (DI) water.

### Preparation of SEBS/MXene Janus Fiber Membrane

Preparation of SEBS Fiber Membrane: SEBS membranes were fabricated via an electrospinning process. A certain amount of SEBS powder was dissolved in tetrahydrofuran (THF) under stirring at 60 °C in an oil bath for 6 h to obtain a fully transparent solution with a final mass fraction of 6%. Subsequently, lithium chloride solution with a concentration ranging from 0.1 to 10 mM was added to the mixture, which was then sonicated for 24 h until a completely clear solution was achieved. The prepared SEBS/LiCl solution was electrospun at a positive voltage of 10 kV and a negative voltage of 2 kV, with a solution flow rate of 1 mL h^−1^. The fibers were collected on a grounded aluminum foil collector, with a fixed distance of 15 cm between the nozzle (23G) and the collector. The resulting SEBS nanofiber membranes were dried overnight at 60 °C under vacuum to remove any residual solvent.

Preparation of MXene: MXene was successfully synthesized through an in situ HF etching method by selectively removing the Al layers from Ti_3_AlC_2_ [33, 34]. Specifically, 1 g of LiF was added to 10 mL of a pre-prepared 9.0 M HCl solution. Under continuous stirring at 35 °C, 1 g of MAX phase precursor (Ti_3_AlC_2_) powder was gradually introduced into the LiF/HCl mixture and etched for 24 h. The resulting product was repeatedly washed with DI water until the supernatant reached a pH > 6, followed by centrifugation at 3500 rpm for 5 min to collect the sediment. To delaminate the Ti_3_C_2_T_x_ layers, the sediment was re-dispersed in 50 mL of DI water and sonicated in an ice bath for 1 h. After subsequent centrifugation at 10,000 rpm, a dark green colloidal suspension of MXene was collected. Previous studies have confirmed that this suspension consists predominantly of monolayer flakes [35, 36]. For subsequent experiments, prepare a concentration of 1.0 mg mL^−1^ MXene solution for later use.

Preparation of SEBS/MXene (SM) Janus fiber membranes: an SEBS fibrous membrane was first prepared via electrospinning, followed by the vacuum-assisted filtration of MXene solution onto the SEBS substrate. By varying the volume of MXene solution (1, 3, and 5 mL), corresponding samples labeled SM1, SM3, and SM5 were obtained. The resulting SM composite membranes had a diameter of 4 cm. For electrical characterization, the membranes were cut into target dimensions with an effective working area of 3 × 3 cm^2^. A sensor was then assembled by attaching two conductive copper electrodes onto the SM Janus fiber membrane.

### Characterization

The microstructure and elemental composition of the samples were characterized using a field emission scanning electron microscope (FESEM, Hitachi SU8010, Japan) equipped with an energy-dispersive X-ray spectrometer (EDX) and a transmission electron microscope (TEM, JEM-2100F, JEOL, Japan). Crystal structures were examined by X-ray diffraction (XRD, Smartlab-SE, Japan) with Cu Kα radiation at a scanning rate of 10° min^−1^ over a 2*θ* range from 10° to 80°. Surface roughness was analyzed via atomic force microscopy (AFM, Dimension Icon, Bruker, Germany). The chemical states of elements were determined by X-ray photoelectron spectroscopy (XPS, Thermo Scientific K-Alpha, USA). Raman spectra were acquired using a Raman spectrometer (DXRxi, USA) with a 532 nm semiconductor laser. Infrared reflectance was measured with a Fourier transform infrared spectrometer (FTIR, Thermo Fisher Scientific Nicolet iS50) equipped with an integrating sphere. Water contact angles (WCAs) were measured using a contact angle goniometer (Dataphysics Contact Angle System Krüss DSA30, Germany), with 3–5 replicates per sample. Thermal images were captured by an infrared thermal analyzer (Hikvision Microvision K20, China). Optical micrographs were obtained using an optical microscope (Axioscope 5, Zeiss, Germany) coupled with a charge-coupled device (CCD) camera. Electromagnetic interference shielding performance was evaluated with an vector network analyzer (N5247A, Malaysia).

### Electrical Characterization

Encapsulation of piezoelectric nanogenerators: To investigate the electrical performance of the SM Janus fiber membrane, a self-powered nanogenerator was encapsulated using copper foil as the conductive layer and polyimide (PI) film as the outer encapsulation layer to prevent MXene degradation. Specifically, the membrane was cut to the target size (effective working area: diameter of 4 cm) and sandwiched between two copper foil electrodes. Electrical wires were soldered to the electrodes to establish connections. The entire assembly was then sealed on both sides with PI films to prolong its service life and enhance the stability of signal transmission.

To visualize the piezoelectric performance of the SM Janus fiber membrane, a nanogenerator system was designed and integrated with a linear motor (HS01-37 166, NTI AG, USA), a digital electrometer (6514, Keithley, USA), and an amplifier (SR570, SRS, USA).

### Sensing Performance Testing

Sensing Performance Testing: The humidity sensing performance was evaluated in a constant temperature and humidity chamber (Kolnans, China,). Systematic investigations were conducted at 25 °C across a relative humidity (RH) range of 20% to 100%. Resistance values were measured using a Keithley 2450 SourceMeter. Written informed consent was obtained from all volunteers prior to the human perception experiments. The sensing signal was characterized by the relative resistance change (Δ*R*/*R*_0_), where ΔR denotes the change in resistance, calculated according to Eq. 1:

1 $$\Delta R{ } = { }\left( {R{ } - { }R_{0} } \right)$$

where *R* is the corresponding instantaneous current at the target humidity, while *R*_0_ is the initial current at 10% RH.

## Results and Discussion

### Design and Preparation of SM Janus Membrane

**Fig. 1:**
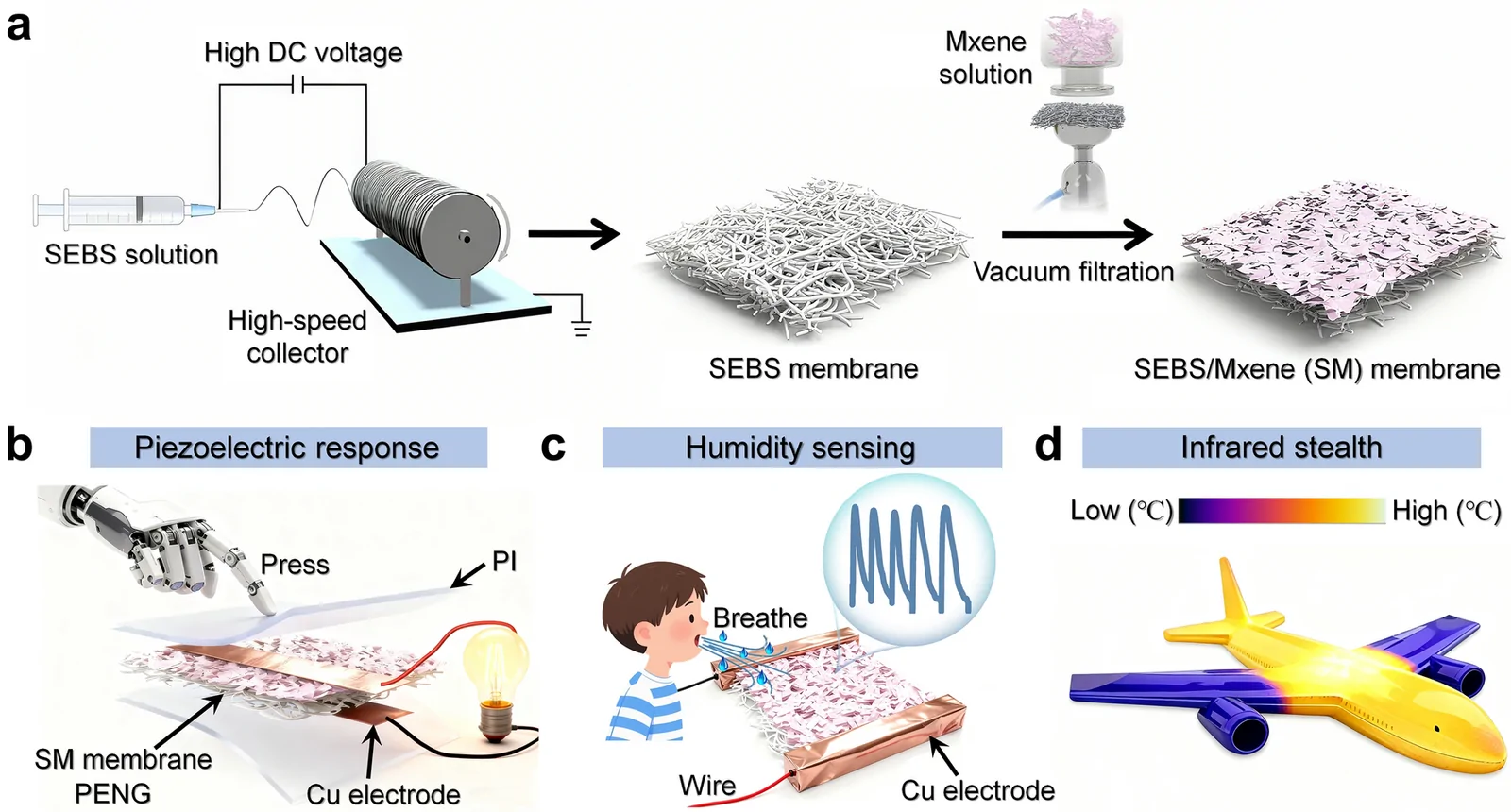
**a** Schematic illustration of the fabrication of SEBS/MXene membrane through electrospun and vacuum filtration. The potential application of SEBS/MXene membrane in **b** piezoelectric response, **c** humidity sensing and **d** infrared stealth

Given the significant challenges inherent in the collaborative integration of multiple functionalities, including energy harvesting, sensing, and environmental interaction within a single material [37, 38], an intelligent Janus membrane was successfully fabricated by combining electrospinning with vacuum-assisted filtration. Figure 1a illustrates the specific fabrication process. Initially, a SEBS nanofiber membrane was prepared via electrospinning as the substrate. This SEBS matrix exhibits excellent biocompatibility and mechanical elasticity, providing robust mechanical support for subsequent electrode layers while adapting to complex skin microenvironments, thereby ensuring close contact with the skin surface in practical applications. Subsequently, a MXene electrode layer was deposited onto the SEBS nanofiber surface using vacuum-assisted filtration. The interconnected MXene nanosheets form continuous and efficient conductive pathways, laying the foundation for the electrical and functional properties of the material. The interfacial adhesion between the SEBS nanofiber membrane and the MXene layer in the SM Janus membrane is a synergistic result of chemical interactions, physical interlocking, and process-induced bonding effects. This multidimensional adhesion mechanism ensures tight integration of the two phases, avoiding delamination even under cyclic mechanical deformation or environmental stimuli. Under external mechanical pressure, the Janus membrane generates a piezoelectric response through interfacial charge separation at the interface between SEBS and MXene. This piezoelectric effect produces a stable voltage output sufficient to illuminate a commercial light bulb (Fig. 1b), demonstrating the material’s practical potential for harvesting energy from the environment. Benefiting from the intrinsic advantages of MXene, including its high specific surface area, abundant surface functional groups (such as –OH and –O), and excellent hydrophilicity, the MXene-modified surface of the Janus membrane efficiently detects changes in environmental humidity and converts physical stimuli into quantifiable electrical signals [39].

In contrast, the hydrophobic SEBS back-layer acts as a moisture barrier, blocking the intrusion of ambient humidity while minimizing interference with the sensing layer [26]. This dual-face structure synergistically enhances the sensitivity, selectivity, and long-term stability of the sensor, enabling reliable humidity monitoring in diverse operating environments (Fig. 1c). Notably, a key advantage of this design is that the piezoelectric output generated by mechanical stimuli can serve as a built-in power source for the humidity sensing module, completely eliminating the need for external power supplies. Furthermore, leveraging the inherent metallic conductivity and plasmonic resonance effects of MXene [29, 40, 41], the Janus membrane exhibits excellent infrared shielding capability (Fig. 1d). This distinctive feature endows it with highly promising application prospects in the military field. For instance, it can effectively evade thermal imaging detection systems through infrared stealth technology, thereby realizing the stealth functionality of aircraft and enhancing their survivability in complex combat environments. To precisely control the MXene layer thickness of the SEBS/MXene Janus membrane and achieve an optimal performance balance for piezoelectric energy harvesting, humidity sensing, and infrared stealth, the strategy must combine quantitative fabrication parameter regulation, real-time structural/property characterization, and performance feedback optimization, centered on the study’s validated optimal MXene deposition volume and the core principle that thickness must balance functional layer continuity, mechanical flexibility, and mass/charge/light transport kinetics.

### Morphology and Composition of SM Janus Membrane

**Fig. 2:**
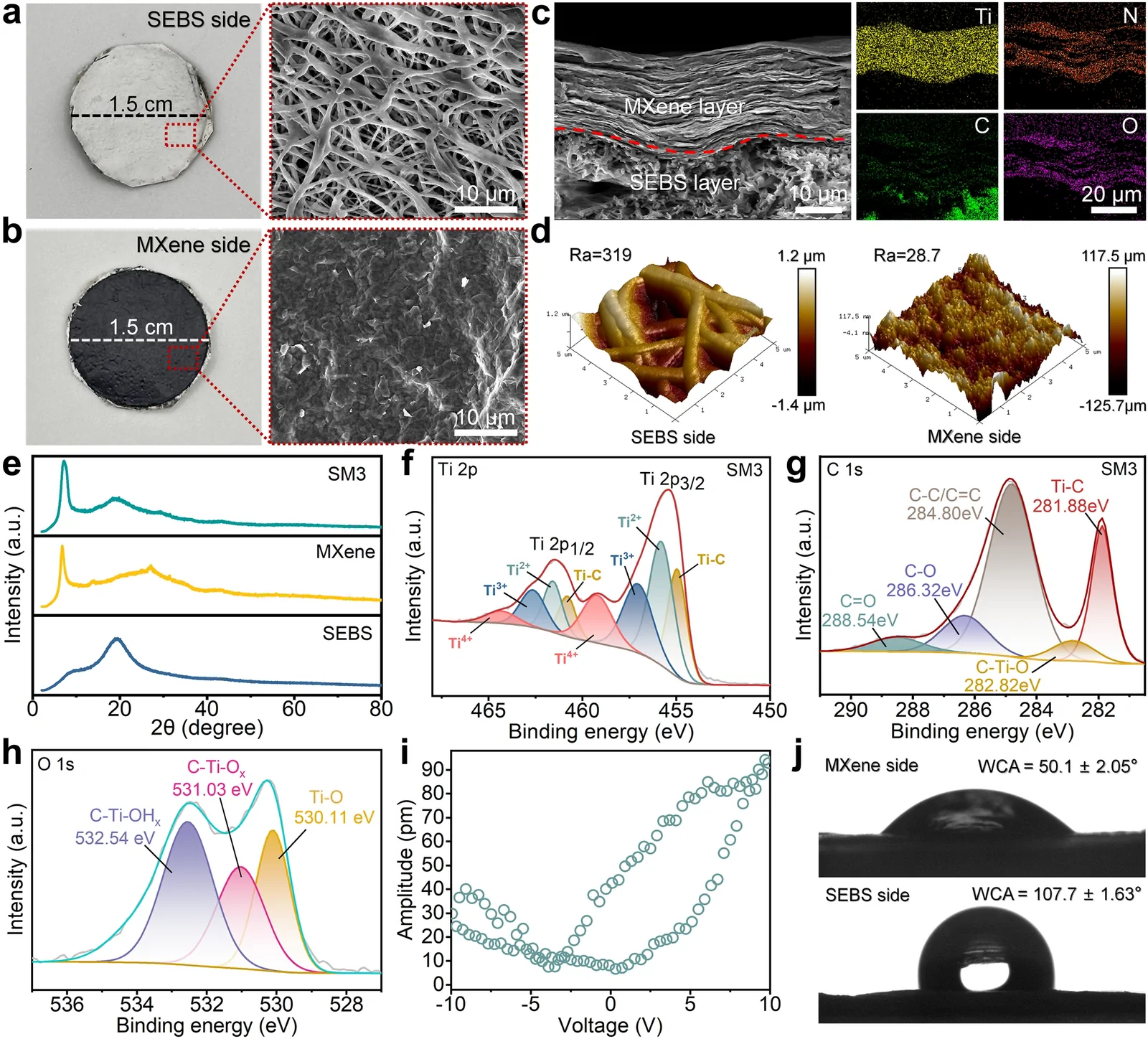
**a** Digital photograph of the SEBS side of SM3 (left) and its corresponding SEM image (right). **b** Digital photograph of the MXene side of SM3 (left) and its corresponding SEM image (right). **c** Cross-sectional SEM image of SM3 along with the corresponding EDS elemental mappings. **d** AFM images of both the SEBS side and the MXene side of SM3. **e** XRD patterns of SM3, MXene, and SEBS. XPS spectrum of **f** Ti 2*p*,** g** C 1*s*, and **h** O 1*s* of SM3. **i** Amplitude–voltage curve of SM3. **j** Water contact angles on the SEBS side and the MXene side of SM3

Figure 2a, b illustrates the unique dual-sided morphology of the as-fabricated membrane, with one side appearing white and the other black. The white side corresponds to the SEBS layer, exhibiting a continuous, uniform, and smooth fibrous structure with an average fiber diameter of approximately 370 nm (Fig. S1). The dual impacts of electrospinning-induced SEBS fiber defects align with three key mechanisms. Mild fiber fusion/adhesion enhances structural stability and fatigue resistance, while excessive fusion reduces elasticity and porosity—SM3’s fiber defects are optimized to balance this trade-off [42]. Flattened fibers and junction fusion increase MXene-SEBS contact area, promoting mechanical interlocking and chemical bonding, which is critical for piezoelectric charge transport and sensing signal stability [43]. Non-uniform fibers and excessive fusion alter porosity, affecting moisture accessibility of humidity sensing and MXene uniformity of infrared stealth [44]. In contrast, the black side consists of the MXene layer, displaying a homogeneous and densely packed surface. The MXene layer exerts a dual synergistic effect on the long-term mechanical stability of the SEBS/MXene Janus membrane under repeated deformation. It reinforces the structural integrity of the SEBS nanofiber substrate while relying on the SEBS’s high elasticity to compensate for its own brittleness, ultimately enabling the membrane to maintain stable mechanical and functional performance after cyclic loading. The cross-sectional field emission scanning electron microscopy (FESEM) image in Fig. 2c further confirms the Janus structure of the membrane. The MXene and SEBS layers are tightly bonded, yet a clearly interfacial boundary is visible due to their morphological differences, indicating that the MXene layer is stacked on top of the SEBS layer in a top-down manner. The SEBS nanofiber membrane is fabricated via a standardized electrospinning process, so its thickness is uniform across all SM samples (SM1/SM3/SM5). The MXene layer thickness varies directly with the filtration volume of MXene dispersion with clear trends. In Fig. 2c, the quantitative thickness of MXene and SEBS layers is approximate at 22 and 62 μm, respectively. The corresponding energy spectrum element mapping also confirms this. Notably, the upper MXene layer is formed by the deposition of a large number of nanosheets. The absence of carbon is due to EDS’s low sensitivity to low-atomic-number elements, signal masking by high-intensity Ti/O/F peaks, and overlap with the SEBS substrate’s carbon signal, complementary XRD/XPS results confirm carbon’s intrinsic presence in MXene. The detected nitrogen originates from external contamination or trace impurities in raw materials, and it is a non-functional trace component that does not affect the MXene layer’s structure or performance. Transmission electron microscopy (TEM) reveals that these nanosheets possess a distinctive two-dimensional (2D) ultrathin layered structure, with lattice fringes spaced at 0.26 nm, consistent with the (100) crystal plane of Ti_3_C_2_T_x_ materials (Fig. S2a) [45]. Atomic force microscopy (AFM) measurements indicate that the MXene nanosheets range in lateral size from 0.1 to 2 μm, while the height profile confirms a thickness of approximately 4 nm, suggesting a monolayer configuration (Fig. S2b).

Benefiting from the structural advantages of MXene nanosheets, its deposition on one side of the SEBS membrane effectively reduces the surface roughness (*R*_*a*_) from 319 to 28.7 μm (Fig. 2d). This is attributed to the filling of inter-fiber voids and the coverage of surface protrusions by MXene nanosheets on the SEBS substrate [46]. To further investigate the influence of MXene deposition amount on the Janus membrane, different volumes of MXene dispersion (1, 3, and 5 mL) were deposited onto the SEBS surface, named as SM1, SM3, and SM5, respectively. Digital photographs (Fig. S3) demonstrate that a 3 mL MXene deposit (SM3) results in a continuous and uniform coating that fully covers the fibrous network and effectively fills the inter-fiber pores. In contrast, insufficient deposition (1 mL, SM1) fails to form a complete and coherent coating, thereby hindering effective electron transport within the fiber membrane and leading to poor electrical conductivity. On the other hand, excessive deposition (5 mL, SM5) tends to cause cracking or delamination of the coating [47]. Therefore, the 3 mL deposition is identified as optimal, as it establishes continuous conductive pathways essential for efficient electron transport, enabling the Janus membrane to exhibit excellent electrical conductivity. This property is critical for high-performance applications such as piezoelectric response, sensing, and infrared shielding. The SEBS-MXene interface is stabilized by Ti–O–C covalent bonding (dominant), hydrogen/electrostatic interactions, and mechanical interlocking, not merely weak non-covalent forces, oxygen-containing functional groups are the core for covalent bond formation. The MXene layer’s durability is evaluated via morphological, cyclic mechanical, and long-term environmental testing; the optimally loaded SM3 sample resists delamination under bending/mild friction, while overloaded SM5 is prone to cracking/delamination. Pronounced surface morphology differences at low MXene loadings stem from the vacuum filtration’s void-filling threshold, monolayer MXene’s high specific surface area, and the porous SEBS substrate; these factors create a critical loading threshold that amplifies morphological changes.

Figure 2e displays the X-ray diffraction (XRD) patterns of SEBS, MXene, and SM3 Janus membranes. The results indicate that the SM3 Janus membrane exhibits characteristic diffraction peaks of both MXene and SEBS components. The three peaks located at 2*θ* = 6.76°, 13.88°, and 27.2° correspond to the (002), (004), and (006) crystal planes of MXene, respectively, suggesting that the aluminum layer in Ti_3_AlC_2_ has been completely removed [48–50], while the peak located at 2*θ* = 19.42° is attributed to the broad diffraction peak of SEBS. Notably, the intensity of this peak decreases with the increase of MXene deposition (Fig. S4) [51]. To further investigate the chemical composition and elemental valence states of the SM3 Janus membrane, X-ray photoelectron spectroscopy (XPS) analysis was conducted. The survey spectrum confirms the presence of C, Ti, F, and O elements in SM3 Janus membrane (Fig. S5). The high-resolution Ti 2*p* spectrum can be deconvoluted into four spin–orbit doublets located at 454.9/460.8, 455.7/461.7, 457.1/462.7, and 459.2/464.4 eV, corresponding to Ti-C, Ti^2+^, Ti^3+^, and Ti^4+^ species (Fig. 2f), respectively [52]. The coexistence phenomenon of multiple titanium valence states clearly reveals the complex chemical state distribution of titanium element in MXene, reflecting the existence of partial oxidation and abundant defect structures on the material surface. In the high-resolution C 1*s* spectrum (Fig. 2g), the characteristic peaks located at 281.88 and 282.82 eV are assigned to Ti–C and C–Ti–O bonds, respectively, which directly confirms the bonding state between carbon and titanium in the MXene lattice and the involvement of oxygen at the interface [51]. The main peak located at 284.8 eV corresponds to C–C/C=C bond in aromatic rings or alkanes, mainly derived from the SEBS matrix, while peaks at 286.32 and 288.54 eV are attributed to C–O and C=O functional groups, indicating a certain degree of oxidation on the polymer phase or MXene surface. The O 1*s* spectrum shows three peaks corresponding to Ti–O (530.11 eV), C–Ti–O_x_ (531.03 eV), and C–Ti–OH_x_ (532.54 eV) (Fig. 2h). The identification of these oxygen-containing groups not only confirms the typical –O and –OH terminations on the MXene surface, but also suggests possible interactions at the interface between SEBS and MXene phases [53].

Subsequently, piezoelectric force microscopy (PFM) was employed to verify the piezoelectric properties of the SM3 membrane. Under alternating voltage, the amplitude and phase responses of SM3 exhibit typical amplitude–voltage butterfly loops and phase hysteresis (Figs. 2i, S6), indicating piezoelectric behavior. The piezoelectricity of the SM Janus membrane is an interfacial heterojunction effect, with the Schottky barrier at the SEBS/MXene interface as the exact source. Mechanical deformation modulates the interfacial built-in electric field, driving directional charge separation, MXene’s conductivity enables efficient charge transport to generate the measurable output. Triboelectricity is negligible, and neither SEBS nor MXene has intrinsic piezoelectricity. The PFM data validate the heterostructure’s interfacial piezoelectric response, consistent with the characterization and mechanistic descriptions. Due to the non-polar nature of SEBS, combined with its fine diameter and high surface roughness [54], the surface exhibits significant hydrophobicity, with a static water contact angle (WCA) of 107.7° ± 1.63°. However, the introduction of MXene, with its abundant oxygen-containing functional groups (–OH, –O, etc.), significantly modulates the surface wettability of the SEBS membrane, reducing the static WCA to 50.1° ± 2.05° and achieving a transition from hydrophobic to hydrophilic (Fig. 2j). This controllable modulation of hydrophilicity/hydrophobicity not only optimizes the interaction interface of the material with the external environment (e.g., moisture, polar media) but also facilitates charge transfer and stress transmission through altered surface wetting states, laying a critical foundation for efficient piezoelectric output and highly sensitive humidity detection.

### Piezoelectric Output Performance of SM Janus Membrane

**Fig. 3:**
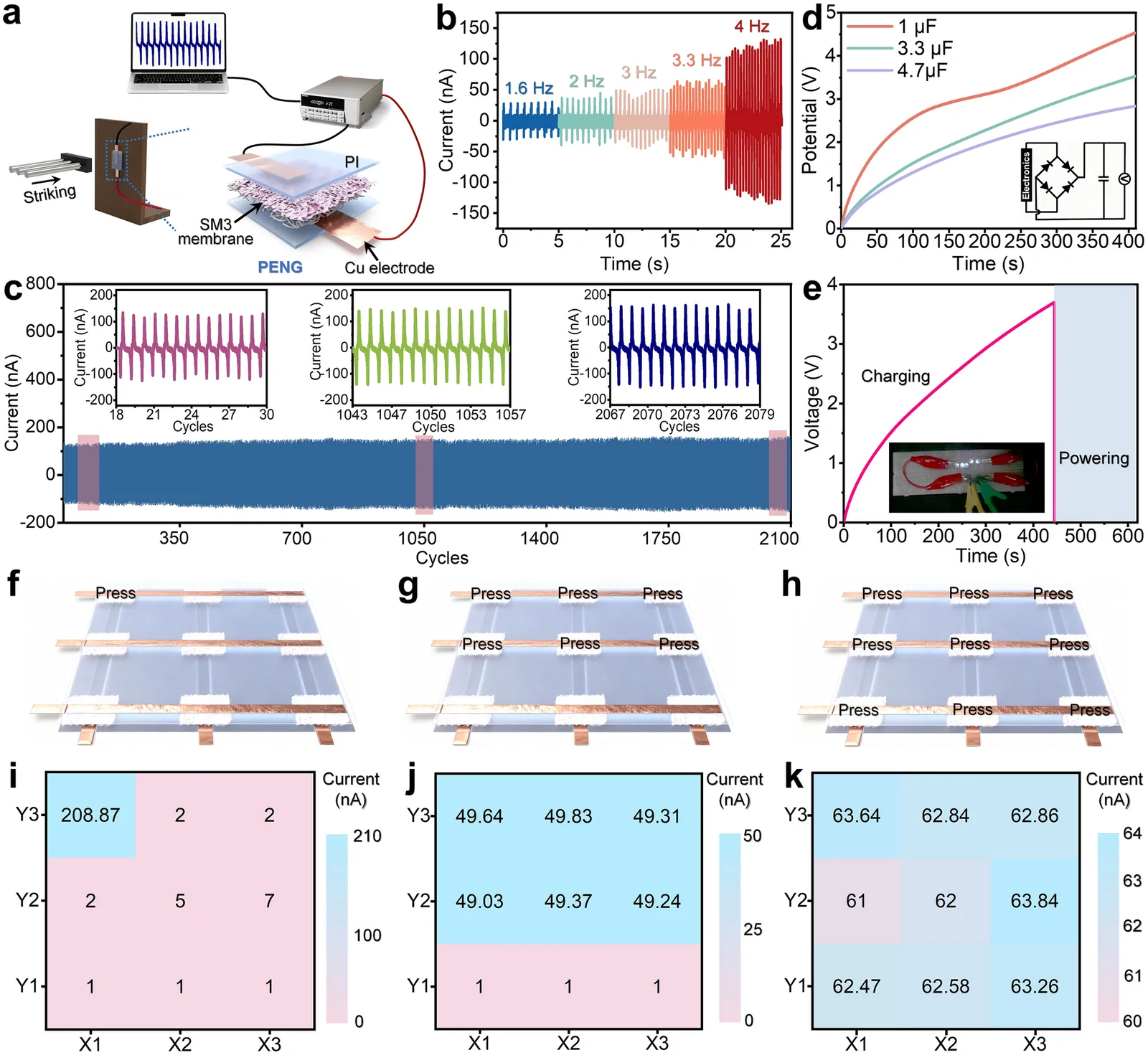
**a** Schematic illustration of the setup for piezoelectric performance measurement (Video S1). **b** Short-circuit current of the SM3-PENG across varying applied frequencies. **c** Durability test of the SM3-PENG. **d** Charging curves of different commercial capacitors, with an inset showing the charging circuit schematic. **e** Charge–discharge profiles of a 3.3 μF commercial capacitor, the inset demonstrates its capability to illuminate a commercial white LED (Video S2). **f–k** Schematic diagram of a 3 × 3 sensor array for pressure location detection and the corresponding results

To evaluate the piezoelectric response of the SM3 Janus membrane, an electromechanical motor with a specified thrust was employed to drive the SM3-based piezoelectric nanogenerator (SM3-PENG), and the testing setup is illustrated in Figs. 3a, S7 and Video S1. The SM3-PENG was fabricated by sandwiching an SM3 Janus membrane between two polyimide (PI) layers for encapsulation to prevent MXene degradation, with copper foils inserted at both the PI-SEBS and PI-MXene interfaces to serve as conductive electrodes. When an external force is applied perpendicular to the surface of the SM3-PENG, the resulting deformation of the SEBS elastomeric substrate induces displacement and interfacial friction among the MXene nanosheets. This process promotes charge separation and interfacial polarization, leading to the alignment of positive and negative charges along the direction of the applied force within the SM3 structure, thereby generating an internal electric potential difference. To balance this potential difference, electrons in the external circuit flow directionally between the PI-SEBS and PI-MXene interfaces via the copper electrodes, producing a measurable electrical signal [55]. Under periodic mechanical impact, the output current and voltage of the SM3-PENG were observed to increase with rising applied frequency, reaching up to 133 nA and 28 V (Figs. 3b, S8), outperforming many performance metrics reported in the literature (Fig. S9). More importantly, the output current remained stable even after 2,100 loading cycles (Fig. 3c), confirming the durability of the device in energy harvesting. To demonstrate that the harvested energy can be rectified and stored, the SM3-PENG was connected to capacitors with different capacitances (1, 3.3, and 4.7 μF) through a bridge rectifier circuit (Figs. 3d, S10). During the 0–400 s charging period, the voltage across the capacitors exhibited a continuous increasing trend, indicating effective storage of piezoelectric energy in each cycle. Interestingly, the stored energy was sufficient to successfully illuminate five commercial white light-emitting diodes (LEDs), as shown in Fig. 3e and Video S2. Therefore, the integration of the SM3-PENG with energy storage modules presents a promising solution to mitigate the issue of frequent battery replacement.

In practical applications, both the magnitude and position of the external forces are critical. To detect the position of the force, the SM3 needs spatial resolution. Therefore, a 3 × 3 sensor array comprising 9 independent SM3 units was designed, and the relevant structural schematic diagram is shown in Figs. 3f–h and S11. When pressure was applied to a single sensor at position X1Y3 using a finger, the output signals of all sensors were recorded. Notably, the stressed sensor exhibited a significantly stronger signal compared to the others (Fig. 3i). Although sensors near the force application points may exhibit some output due to the inherent rigidity of copper foil or PI film, their signals are significantly smaller compared to those from the force application points. This demonstrates that by monitoring the output signals from the sensor array, the location of the applied pressure can be detected. In addition, the array configuration enables simultaneous detection of multiple pressure points. As shown in Fig. 3j, when pressure was applied to the six sensors in the first two rows, their current signals were markedly higher than those from the three sensors in the third row. A similar response pattern was observed when all nine sensors were pressed simultaneously (Fig. 3k). Such differential signaling makes it feasible to accurately identify the locations of applied forces. Hence, in scenarios involving multipoint or large-area loading, each SM3 unit in the array can respond independently, allowing simultaneous monitoring of multiple stressed regions through analysis of their individual signals. To minimize signal crosstalk between adjacent units in the 3 × 3 SEBS/MXene Janus membrane sensor array, especially when pressure is applied to overlapping/nearby regions, targeted structural, material, and fabrication optimizations are required, building on the intrinsic properties of the SM Janus membrane and addressing the core crosstalk causes of mechanical stress propagation, electrical leakage, and substrate rigidity. The piezoelectric energy output of voltage, current, and power of the SM3-PENG scales with the effective working area of the SEBS/MXene Janus membrane in a directionally dependent and area-correlated manner, governed by the fundamental mechanisms of interfacial charge separation of SEBS-MXene interface and charge transport through the MXene layer. While the study does not report explicit multi-area quantitative data, the scaling behavior can be deduced from its piezoelectric mechanism, structural design, and experimental characterizations, with clear physical and structural constraints defining the scaling trend. In summary, the SM3-PENG exhibits remarkable performance in both piezoelectric energy harvesting and arrayed tactile positioning. These results not only confirm its potential as a flexible self-powered sensing unit, but also lay a solid foundation for the development of fully self-sustaining humidity sensing systems without the need for an external power supply.

### Humidity Sensing Performance of SM Janus Membrane

**Fig. 4:**
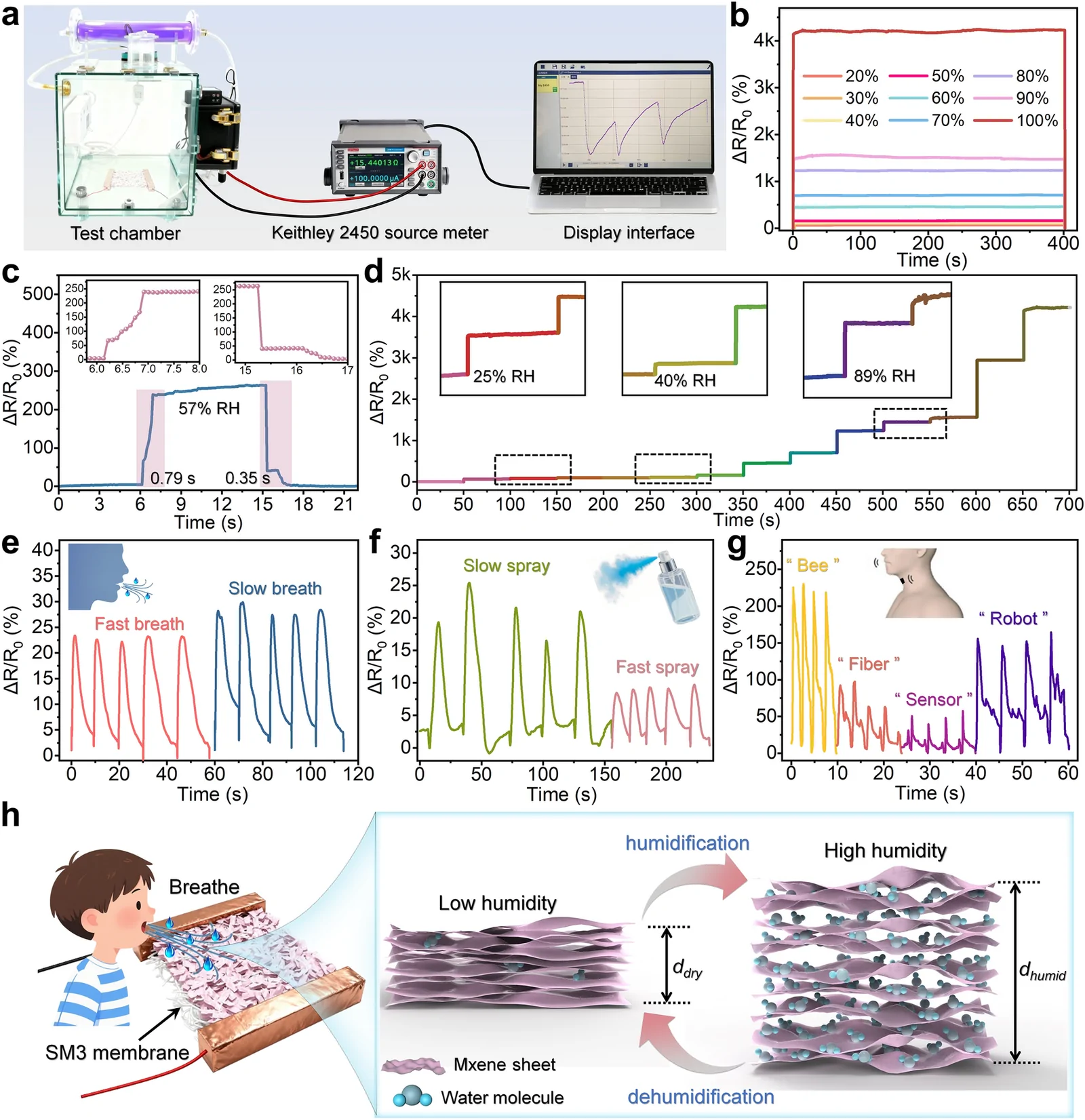
**a** Schematic representation of the dynamic humidity response testing system for SM3. **b** Long-term response/recovery curves of the SM3 under different humidity conditions. **c** Response/recovery curve of SM3 sensor in 57% RH. **d** Continuous step-response curves of SM3. The SM3 sensor demonstrates electrical resistance responses under multiple realistic conditions, including **e** human oral respiration, **f** atomizer spray exposure environment (Video S3), and **g** pronunciation of different syllables. **h** Schematic diagram of the SM3 humidity sensing mechanism

Benefiting from the exceptional hydrophilicity, high electrical conductivity, and large specific surface area of MXene materials, the SM3 Janus membrane exhibits strong potential as humidity sensors. However, conventional humidity sensors face significant limitations in practical applications, particularly their reliance on external power supply, which not only increases operational costs but also restricts their use in flexible scenarios [56]. In contrast, the SM3 Janus membrane can effectively overcome these drawbacks. Under external pressure, it generates a stable piezoelectric response, and the output electrical signals can be stored via a capacitor, thereby enabling self-powered operation of the humidity sensor without the need for additional power supply equipment. Here, the humidity sensing performance of the SM3 Janus membrane was evaluated using a dynamic testing system (Fig. 4a). Figure 4b illustrates long-term response/recovery curve of the SM3 sensor under conditions ranging from 20% to 100% RH. It should be noted that 20% RH was set as the minimum detection threshold because humidity below this value result in unstable electrical signal outputs, similar to environmental noise interference [57]. As shown in Fig. 4b, the SM3 sensor exhibits high sensitivity and stability in response to humidity variations. The highest response value was observed under 100% RH conditions, indicating that the sensing material adsorbs more water molecules under high-humidity conditions, leading to a greater change in electrical resistance. More importantly, even after three months of exposure to ambient atmospheric conditions, the SM3 sensor maintained a high level of electrical signal output (Fig. S12), demonstrating excellent long-term stability. This is attributed to PI encapsulation and the hydrophobic SEBS layer that protect the copper electrode from oxidation. In addition, the Ti–O–C covalent bonding and mechanical interlocking stabilize the SEBS-MXene interface. The self-powered design that avoids external bias-induced degradation and the intrinsic chemical stability of MXene. The SM3 sensor’s long-term performance stability is evaluated based on three core quantitative criteria. The relative signal attenuation (*ΔR*/*R*₀) ≤ 15%, response/recovery time prolongation ≤ 43% (recovery) and ≤ 27% (response), and baseline resistance drift ≤ 20%. These criteria ensure the sensor retains functional utility for humidity sensing applications after three months of ambient storage, confirming its resistance to MXene oxidation-induced degradation, enabled by the Janus structure’s protective effect. Additionally, the humidity sensor exhibits fast response and recovery characteristics. At 57% RH, the response and recovery times were measured to be 0.79 and 0.35 s, respectively (Fig. 4c). Figure 4d shows the continuous step-response curve of the sensor. As humidity gradually increased, the SM3 sensor consistently exhibited rapid responses, confirming its ability to adapt to dynamic changes in external humidity. Overall, the humidity‑sensing performance of SM3, whether in terms of response/recovery time or the upper detection limit, surpasses most values reported in the literature (Fig. S13).

Leveraging its fast humidity response characteristics, the SM3 sensor can also be used for real-time identification of oral breathing. Since exhaled breath contains a large amount of water vapor, placing the humidity sensor near the oral or nasal cavity generates detectable electrical signals (Fig. 4e). This not only enables non-contact and comfortable physiological monitoring but also holds potential for distinguishing breathing patterns, thereby supporting future respiratory correction therapies [58]. In addition, the sensing performance of the device was tested under different nebulizer spray frequencies, and the sensor successfully detected distinct signal patterns (Fig. 4f and Video S3). More interestingly, the SM3 sensor can also recognize articulation characteristics of different syllables by monitoring oral airflow during speech (Fig. 4g).

The humidity sensing mechanism of the SM3 Janus membrane was further analyzed. As shown in Fig. 4h, the sensing material consists of MXene and SEBS, with MXene containing abundant hydrophilic functional groups. In low-humidity environments, the two-dimensional MXene nanosheets are tightly stacked, resulting in low electrical resistance. Conversely, in high-humidity environments, adsorbed water molecules form a thin water film on the MXene side of the SM3 Janus membrane. Under an external electric field, hydronium ions (H_3_O^+^) generated from the water film act as charge carriers, thereby enhancing the conductivity of the composite membrane. However, when water molecules penetrate into the interior of the composite membrane, they induce swelling of MXene, leading to an increased interlayer spacing and weakened effective contact between MXene nanosheets, which ultimately raises the electrical resistance of the composite membrane [59–63]. Simultaneously, the hydrophobic SEBS backing layer serves as a moisture barrier, preventing environmental humidity from penetrating the sensing layer and minimizing interference. This Janus structure synergistically enhances the sensitivity, selectivity, and long-term stability of the sensor, enabling reliable humidity monitoring across diverse working environments. The SM3 sensor is harmless to the human body for real-time oral breathing identification, with safety and biocompatibility guaranteed by the intrinsic non-toxicity and biocompatibility of SEBS, MXene, toxic residues/hazardous byproducts, a non-invasive, physically passive sensing mechanism with no chemical/electrical stimulation of human tissues. The Janus configuration solves the core limitations of homogeneous SEBS/MXene composites of slow mass transport, inefficient charge separation, non-uniform functional performance, cross-interference, and traditional layered integration of poor interfacial compatibility, and delamination. This validation confirms that the SM Janus membrane is a pioneering material platform for synergistic multifunctional integration, and the Janus asymmetric structure is the critical enabler of its superior performance.

### Infrared Stealth and Camouflage Properties of SM Janus Membrane

**Fig. 5:**
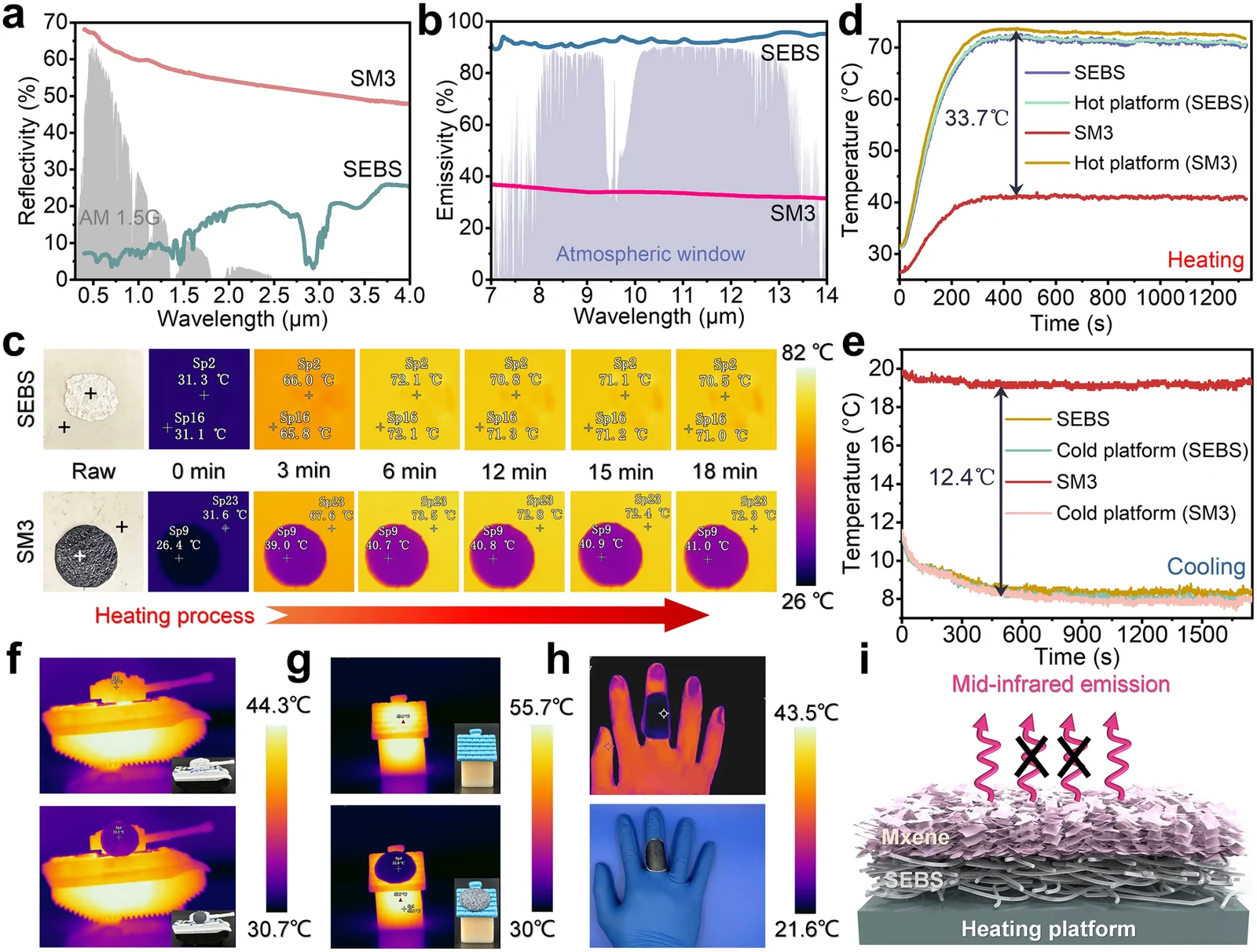
**a** Reflectance spectra in the ultraviolet–visible–near-infrared range and **b** emissivity spectra in the mid-infrared range of SEBS and SM3. **c** Infrared thermal images of SEBS and SM3 obtained on an 80 °C hot platform. **d** The corresponding temperature–time curves. **e** Temperature–time curves of SEBS and SM3 on an 8 °C cold platform. **f–h** Infrared thermal images of SM3 partially covering different target regions. **i** Schematic illustration of the infrared stealth mechanism of SM3

Traditional infrared stealth coatings mainly utilize metallic materials. However, their high density, susceptibility to corrosion, and high glossiness characteristics may result in incompatibility with visible or near-infrared light [64]. Notably, MXene materials exhibit low emissivity in the infrared region due to their unique band structure and high carrier density, which helps to suppress the emission of surface infrared radiation [29, 65]. In addition, the composite structure of MXene and SEBS membrane can significantly hinder solar thermal energy conversion through sunlight reflection, while maintaining the low infrared emissivity characteristics of MXene. As shown in Fig. 5a, the SM3 membrane demonstrates an average solar reflectance of 55%, substantially higher than that of SEBS membrane. Meanwhile, SM3 maintains a low infrared emissivity of approximately 32%, much lower than that of SEBS membranes (Fig. 5b). The phenomenon that the emissivity of SM5 (5 mL MXene dispersion) is higher than that of SM3 is essentially determined by the structural defects of the MXene layer induced by overloading, the destruction of the low-emissivity mechanism, and the synergistic effect of the Janus membrane structure, consistent with the latest research findings in the field [66, 67]. It is worth noting that the amount of MXene deposition influences the emissivity of SM membrane (Fig. S14). Overall, SM membrane is well fit for low-emissivity infrared stealth with synchronous solar thermal energy management.

The infrared stealth performance of SM membranes was evaluated based on the temperature difference between the radiative temperature of the sample surface and the hot/cold target. Firstly, an 80 °C hot platform was used to simulate a hot target, and all samples placed on it (Fig. S15a). Infrared thermal imaging was employed to record the temperature changes of the sample surface and the hot target during heating. Results indicate that all SM membranes exhibited lower surface radiative temperatures compared to the SEBS membrane (Fig. S15b, c). Among them, SM3 showed the lowest surface radiative temperature (40.8 °C), which is 33.7 °C lower than the hot target temperature (Fig. 5c, d), demonstrating its suitability for extreme outdoor environments such as summer or desert conditions. Additionally, SM membranes also exhibit stealth capabilities for cold objects. A cold stage maintained at 8 °C was employed to simulate a cold target, with all samples placed thereon (Fig. S16a). As shown in Fig. S16b, c, all SM membranes displayed higher surface radiative temperatures compared to the SEBS membrane. Specifically, SM3 achieved a maximum surface radiation temperature of 19.2 °C, close to room temperature and 12.4 °C higher than the cold target temperature (Fig. 5e), highlighting its excellent performance in mild spring and autumn climates. More importantly, compared to many materials reported in the literature, the SM membrane in this study demonstrate competitive performance characteristics (Fig. S17). Ma et al. [27] reported the liquid metal/SEBS fibrous membrane with blended electrospinning, which exhibit the energy harvesting of piezoelectric (15 V/70 nA) with 1000-cycle stability, humidity sensing (*t*_res = 2.0 s, *t*_rec = 1.3 s; *ΔR*/*R*₀ = 60 @100% RH), infrared performance (*ε* = 45%; *R*_solar = 35%; hot target cooling: 20 °C). Ye et al. [30] developed MXene/graphene oxide core–shell fibers with core–shell electrospinning, which exhibit the energy harvesting of triboelectric (10 V/50 nA) with 800-cycle stability, humidity sensing (*t*_res = 1.7 s, *t*_rec = 1.1 s; *ΔR*/*R*₀ = 75 @100% RH), and infrared performance (*ε* = 36%; *R*_solar = 50%; hot target cooling: 28 °C).

To further verify the stealth effect of SM membranes, they were attached to the surfaces of preheated house and tank models. As shown in Fig. 5f, g, the areas covered with SM3 membrane were almost indistinguishable from the background. More importantly, due to its outstanding flexibility, the SM3 membrane can be bent to conform to the surface of a finger, effectively reducing the surface radiation temperature (Fig. 5h) and enabling the covered area to fully blend with the background, thereby achieving the stealth effect. Figure 5i illustrates the infrared stealth mechanism of SM membranes. By leveraging the low-emissivity MXene layer, SM membranes ultimately exhibit low infrared radiation intensity, thus achieving excellent infrared stealth performance. The thickness of the MXene layer, tailored by depositing 1, 3, and 5 mL of MXene dispersion to form SM1, SM3, and SM5 samples, exerts a dose-dependent, synergistic/antagonistic influence on the piezoelectric output, humidity sensing sensitivity, and infrared stealth performance of the SEBS/MXene Janus membrane, creating a critical trade-off that is optimized at the 3 mL deposition (SM3), the thickness that balances continuous functional layer formation, mechanical flexibility, and interfacial compatibility. The SEBS/MXene (SM) Janus membrane exhibits good intrinsic environmental reliability for short-to-medium-term exposure, with core functionalities remaining stable under ambient conditions. However, targeted protective coating/encapsulation is necessary for long-term or harsh environmental applications, as the MXene layer and SEBS-MXene interface face degradation risks that gradually compromise performance without protection.

## Conclusion

In summary, this study successfully fabricated SEBS/MXene (SM) Janus fiber membranes by combining electrospinning technology with vacuum-assisted filtration, achieving the collaborative integration of multiple key functionalities. The high elasticity and hydrophobicity of the SEBS electrospun membrane perfectly complement the high electrical conductivity, hydrophilicity, and low emissivity of the MXene layer. Results show that the SM Janus membrane generates stable electrical signal output under periodic mechanical pressure. Both output voltage and current increase synchronously with the elevation of load frequency, reaching up to 28 V and 133 nA, which can achieve efficient charging and discharging of capacitors. The synergistic effect of the hydrophilic MXene layer and the hydrophobic SEBS layer endows the sensor with high sensitivity characteristics, with response and recovery times of only 0.79 and 0.35 s, respectively.

At the same time, the sensor can detect human respiration (distinguishing between fast and slow breathing), humidity changes from sprays, and syllable recognition in speech. Most notably, the electrical signal generated by piezoelectricity can provide energy for humidity sensing without the need for additional power equipment. In addition, benefiting from the porous structure of the SEBS membrane and the infrared radiation shielding capability of the MXene layer, the SM Janus membrane exhibits a low infrared emissivity of 32%. It can reduce the radiant temperature of an 80 °C target to 40.8 °C and increase that of an 8 °C target to 19.2 °C, demonstrating excellent infrared stealth performance. The biocompatibility stems from the intrinsic material properties of SEBS and MXene, the structural design of the Janus membrane, and the absence of toxic/irritant components in its fabrication. The experimental data provide conclusive evidence of functional orthogonality. Minor interference at extreme conditions is identified and mitigated by the reported design, with clear future optimization strategies to further enhance performance robustness. In summary, the SM Janus membrane represents a successful example of synergistic multifunctional integration with intentional orthogonality, addressing the core challenge of inter-function interference in flexible intelligent systems and promising potential for multifunctional applications in complex scenarios.

## Supplementary Information

Below is the link to the electronic supplementary material.

Supplementary Material 1

Supplementary Material 2

Supplementary Material 3

Supplementary Material 4
