# Supplementary material for: Multi-Responsive SEBS/MXene Janus Membranes Enabling Piezoelectric Energy Harvesting, Humidity Sensing, and Infrared Stealth
Source: Nanomicro Lett. 2026 May 11;18:365. doi: 10.1007/s40820-026-02184-x (PMC13161438; doi:10.1007/s40820-026-02184-x)
Supplement: Supplementary file 4 — Supplementary Material 4 [file 40820_2026_2184_MOESM4_ESM.docx]

Supporting Information for

**Multi-Responsive SEBS/MXene Janus Membranes Enabling Piezoelectric Energy Harvesting, Humidity Sensing, and Infrared Stealth**

Weiwen Wang^1^, Hong Ma^1^, Lun Zhang^2^, Jihai Zhang^1,^ *, Aimin Zhang^1,^ *

^1^ State Key Laboratory of Advanced Polymer Materials, Polymer Research Institute, Sichuan University, Chengdu 610065, P. R. China

^2^ Hubei Three Gorges Laboratory, Hubei,Yichang 443007, P. R. China

^*^Corresponding authors. Email: [zhangjihai@scu.edu.cn](mailto:zhangjihai@scu.edu.cn) (Jihai Zhang); [zhangaimin@scu.edu.cn](mailto:zhangaimin@scu.edu.cn) (Aimin Zhang)

**Supplementary Figures and Videos**


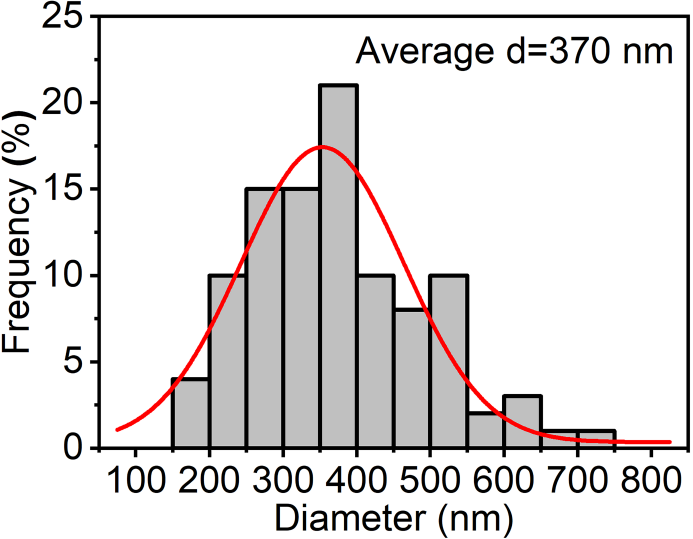


**Fig. S1** The calculated fiber diameter distribution curve


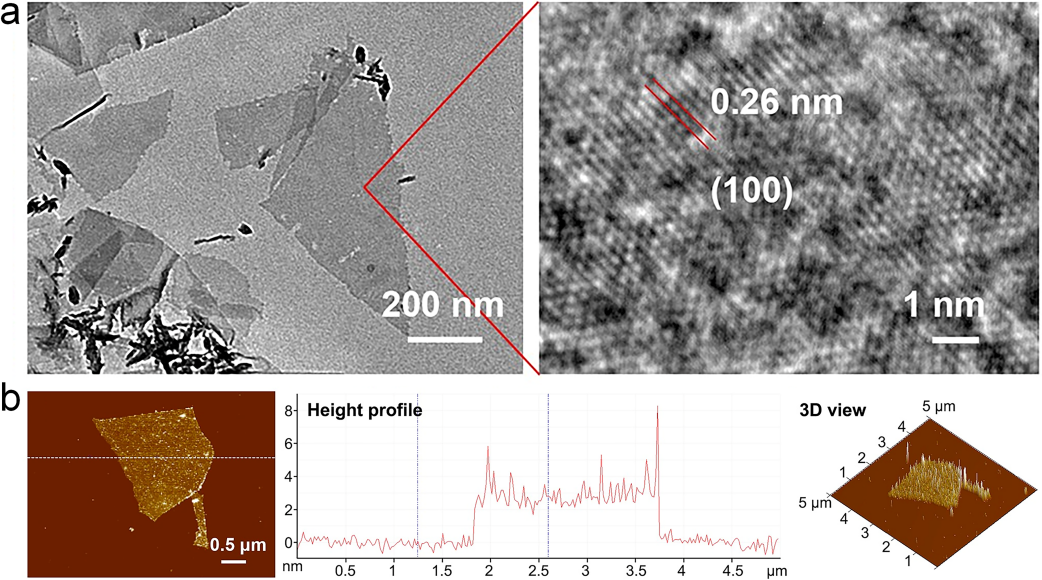


**Fig. S2** **a** TEM image of Ti_3_C_2_T_x_ nanosheets with different magnifications. **b** AFM image of Ti_3_C_2_T_x_ nanosheets and corresponding height profile of crossed line


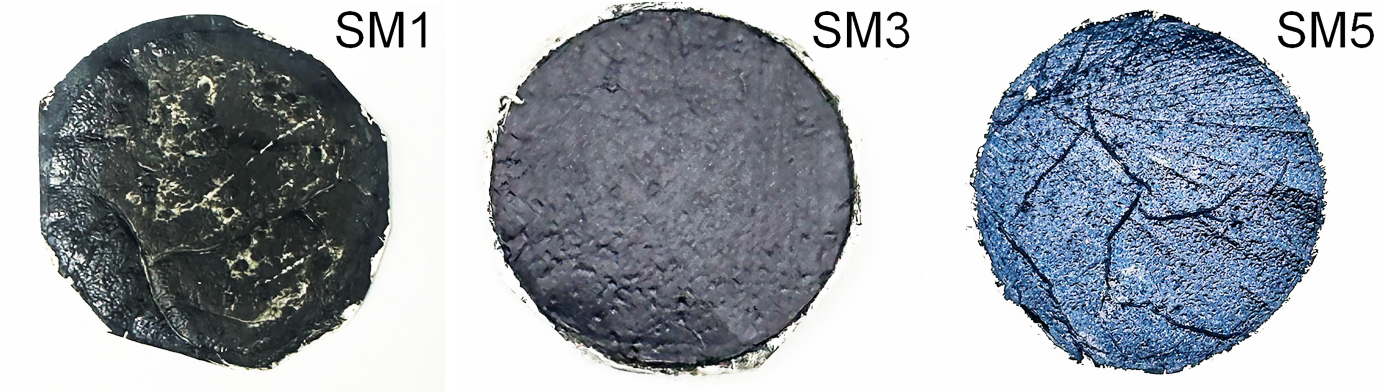


**Fig. S3** Digital photos of SM1, SM3, and SM5


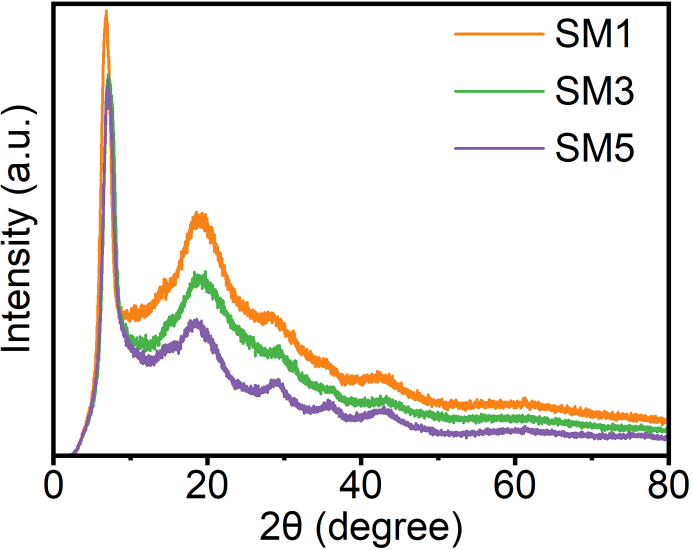


**Fig. S4** XRD patterns of SM1, SM3, and SM5


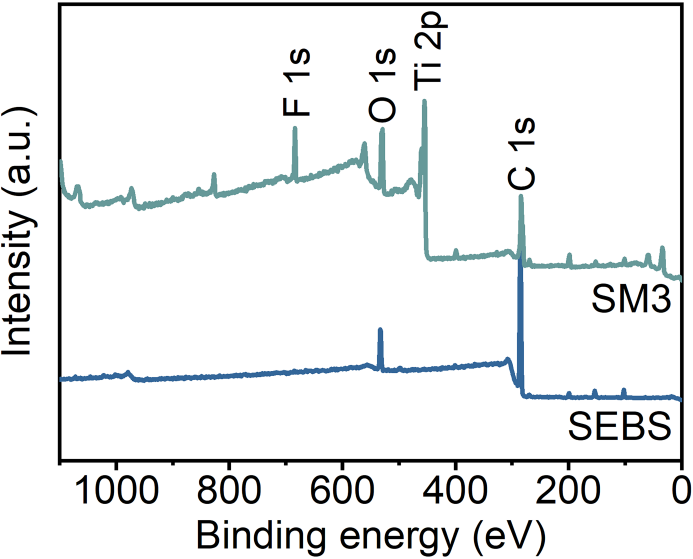


**Fig. S5** The XPS survey spectrum of SEBS and SM3


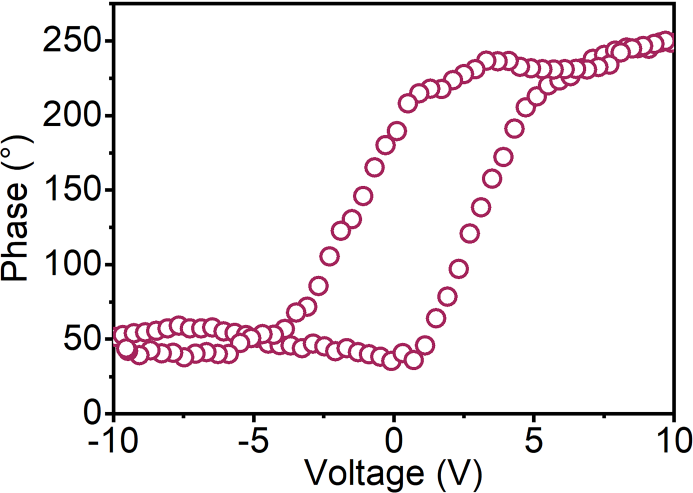


**Fig. S6** Phase-voltage curve of SM3


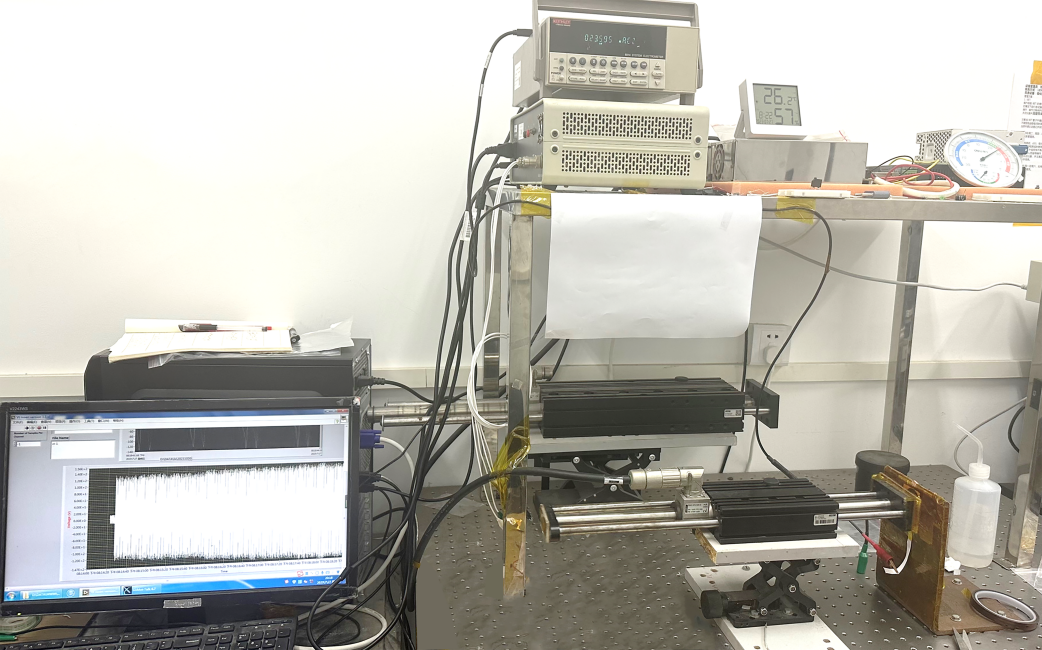


**Fig. S7** Piezoelectric Performance Measurement Device


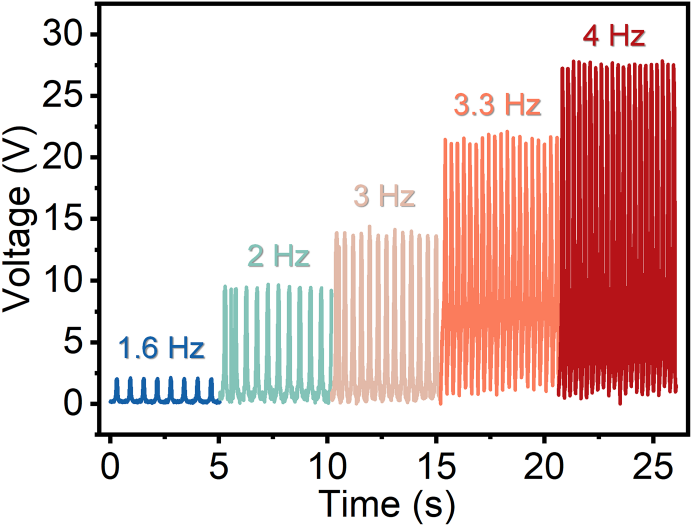


**Fig. S8** Open-circuit voltage of the SM3-PENG across varying applied frequencies


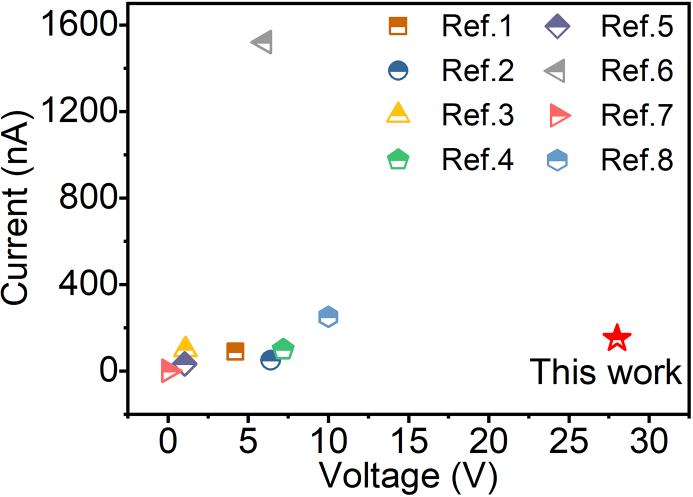


**Fig. S9** Comparison of piezoelectric performance between SM and other materials


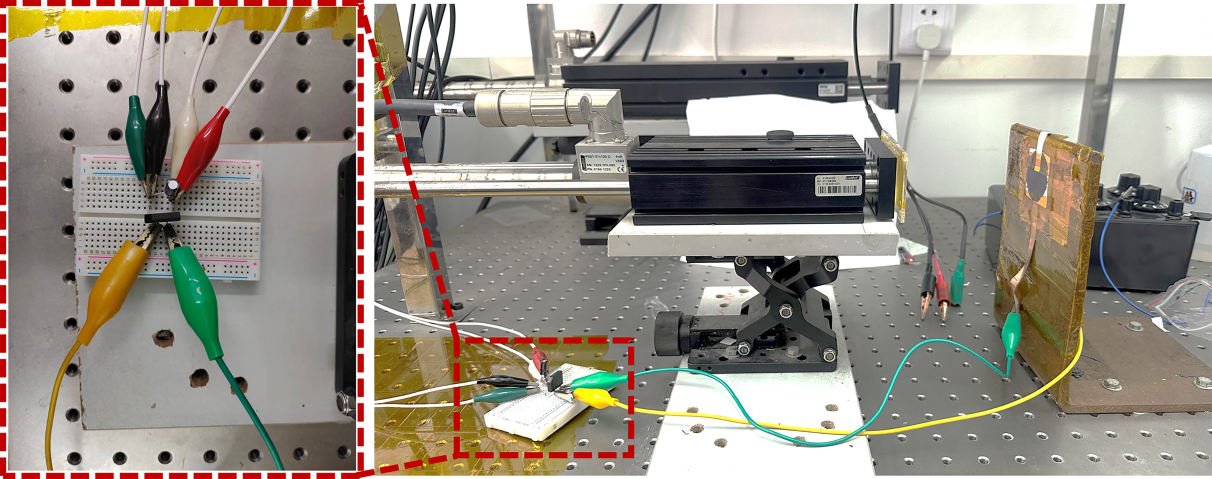


**Fig. S10** Charging device for commercial capacitors


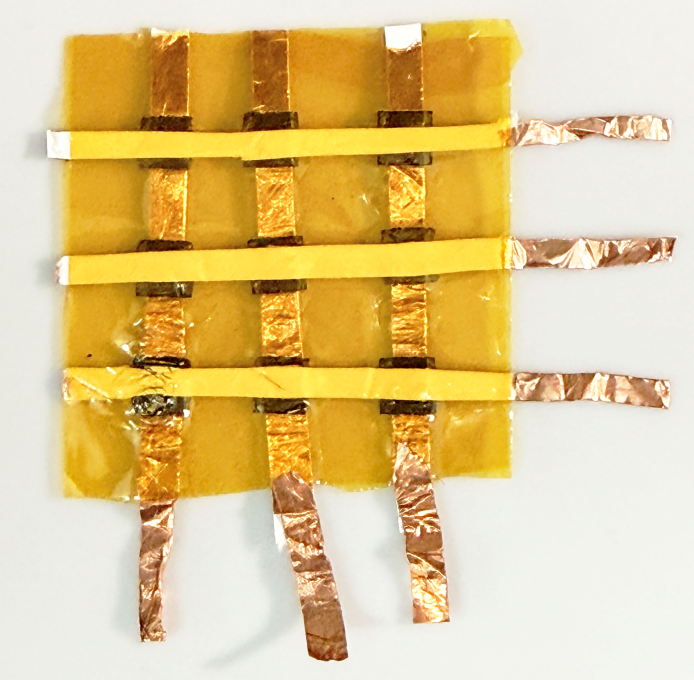


**Fig. S11** 3×3 Sensor Array Device for Pressure Position Detection


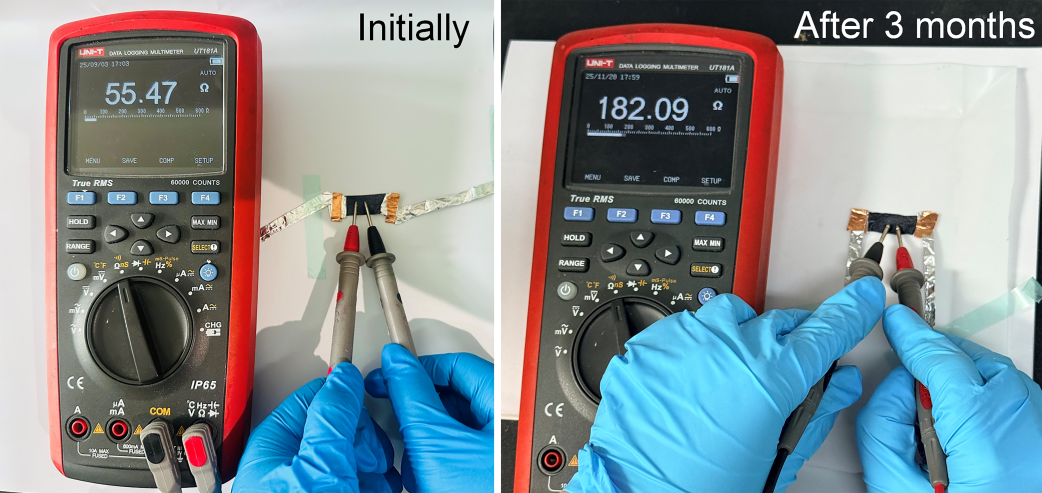


**Fig. S12** Resistance response of SM3 before and after exposure to the environment for three months


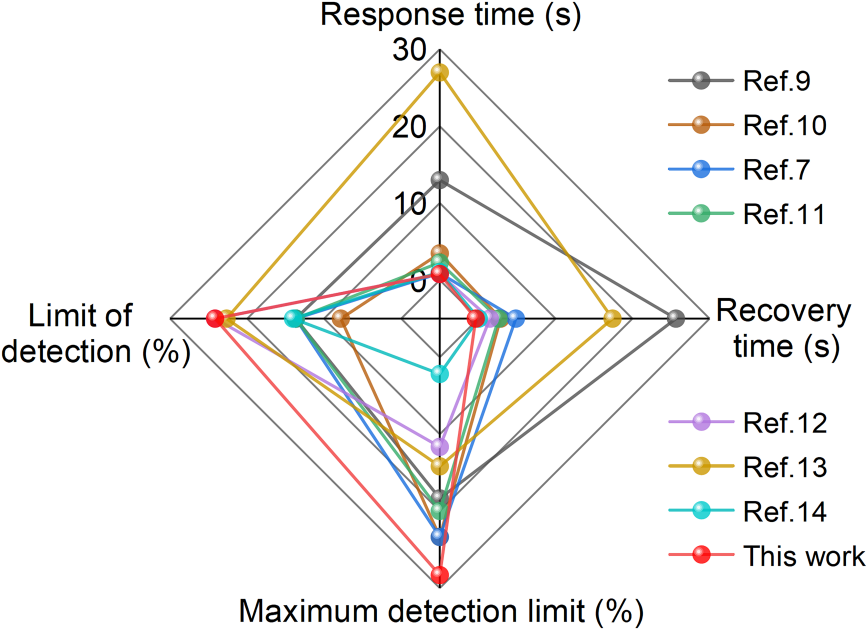


**Fig. S13** Comparison of humidity sensing performance of SM with other materials


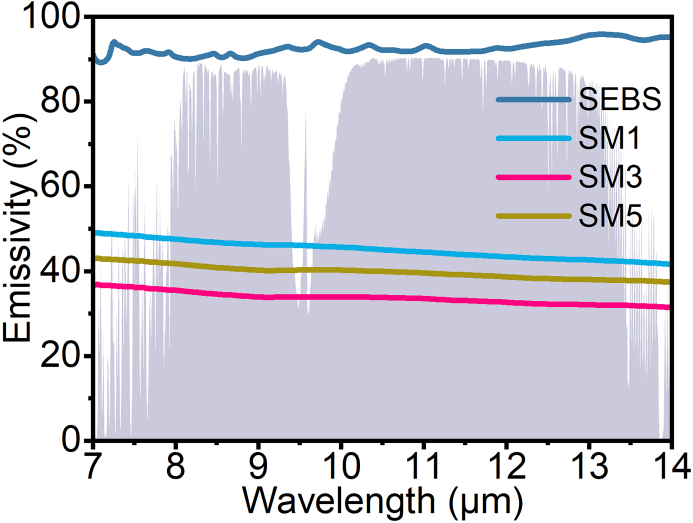


**Fig. S14** Emissivity spectra in the mid-infrared range of SEBS, SM1, SM3, and SM5


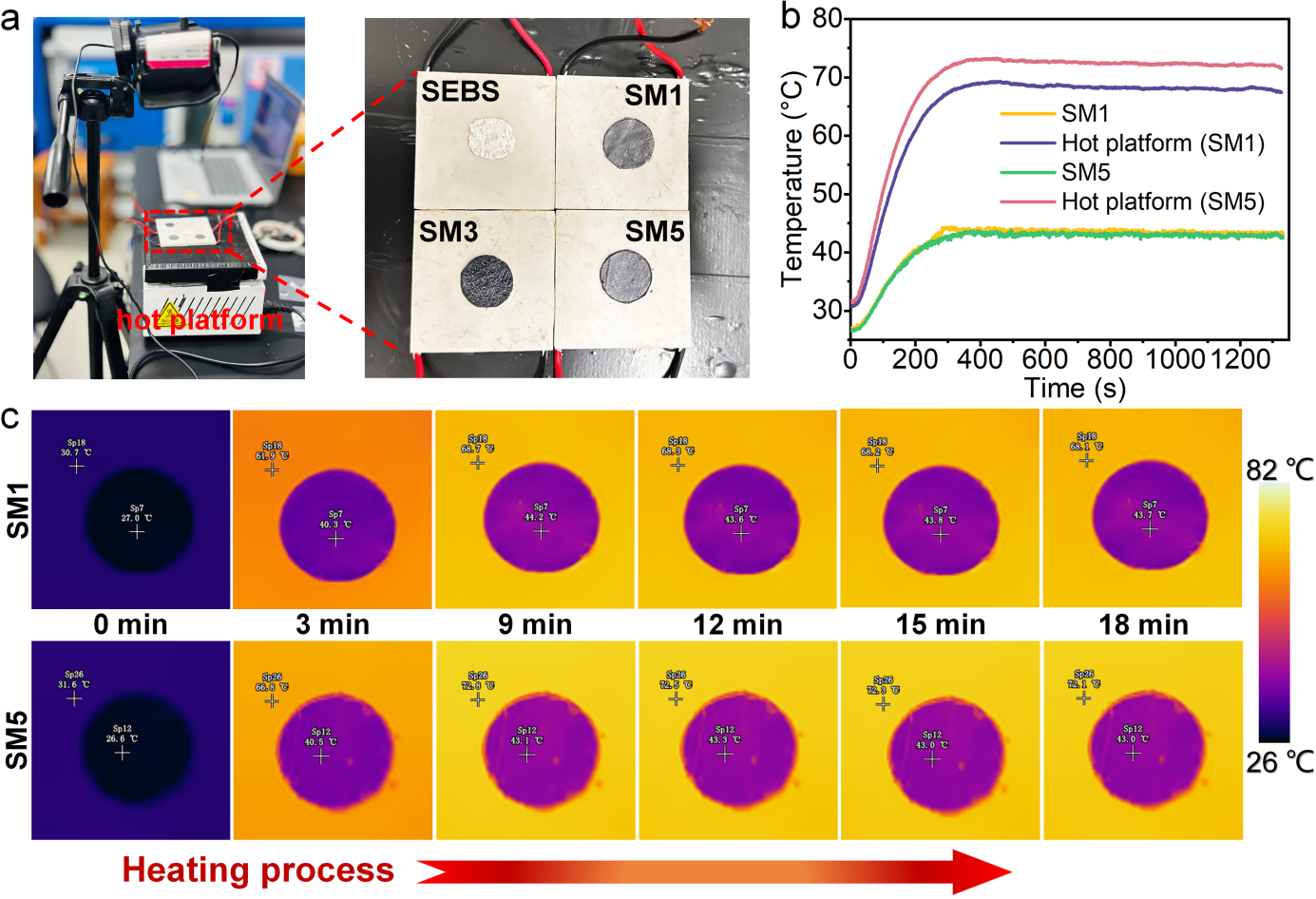


**Fig. S15** **a** Experimental setup for testing the infrared stealth performance of SM on a hot platform. **b** Temperature-time curves of SM1 and SM5 obtained on an 80°C hot platform. **c** The corresponding infrared thermal images.


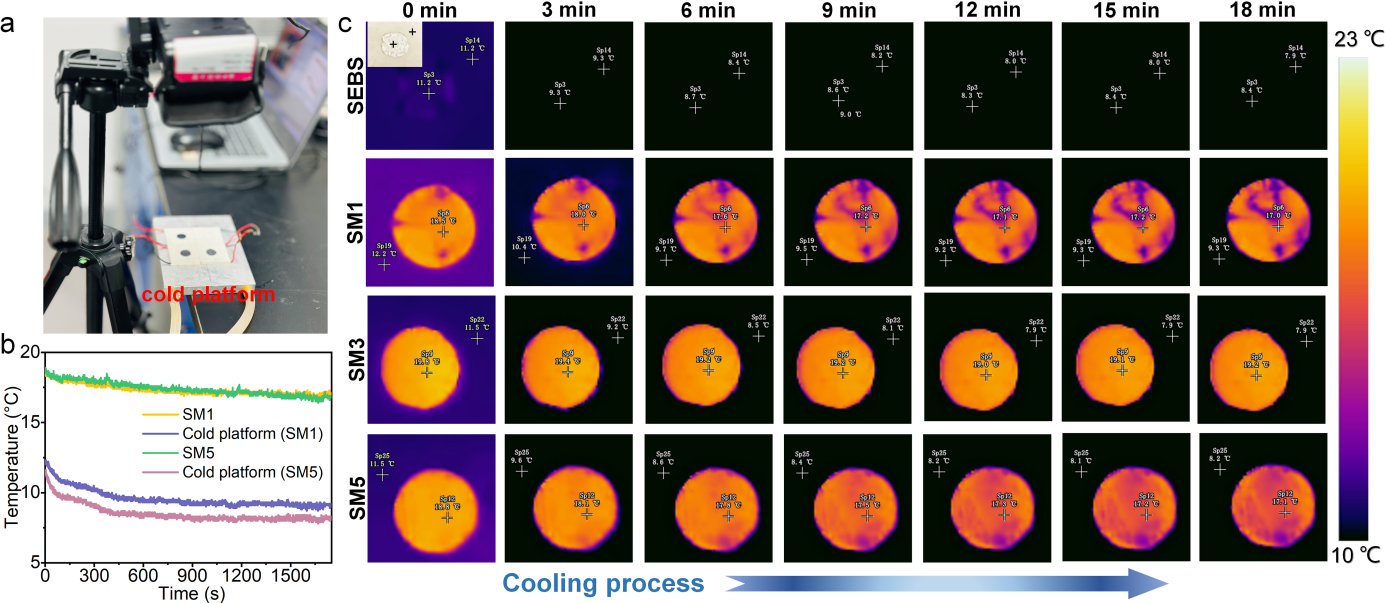


**Fig. S16** **a** Experimental setup for testing the infrared stealth performance of SM on a cold platform. **b** Temperature-time curves of SM1 and SM5 obtained on an 8°C cold platform. **c** The corresponding infrared thermal images


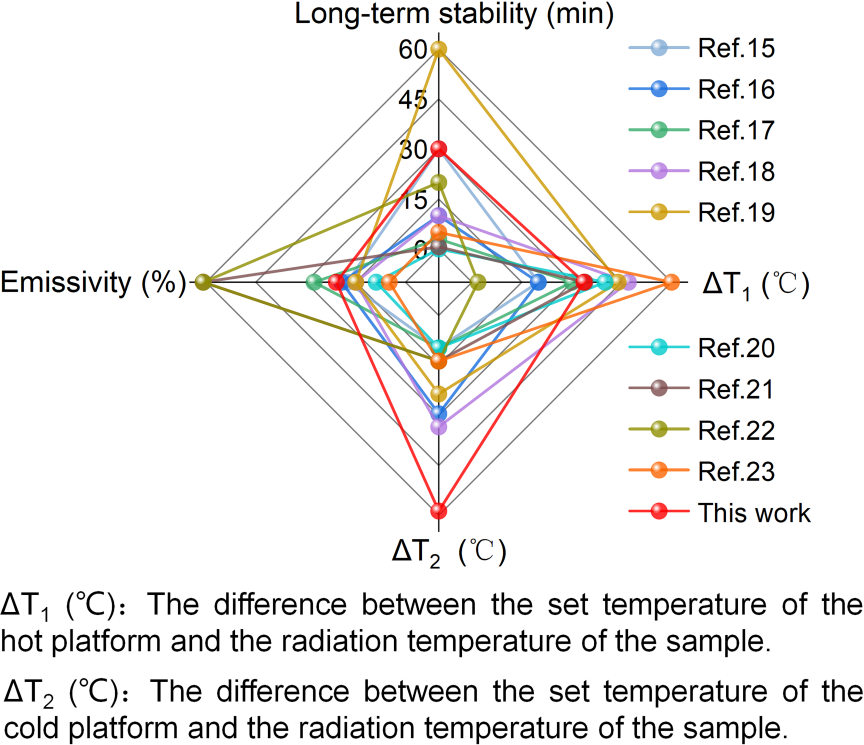


**Fig. S17** Comparison of infrared stealth performance of SM with other materials

**Supporting Movies**

**Video S1** Demonstration of piezoelectric device signal gener.

**Video S2** Demonstration of SM3-PENG driving commercial LED illumination via linear motor impact.

**Video S3** Testing of device sensing performance at different nebulizer spray frequencies.

**Supplementary References**

1. Y. Zu, Y. Sun, C. Wang, Y. Ji, Y. Zhang et al., MXene-driven configurational remodeling of PVDF microstructures: a pathway to high piezoelectricity nanofibers for human motion recognition. Chem. Eng. J. **519**, 164939 (2025). <https://doi.org/10.1016/j.cej.2025.164939>
2. Y. Fu, J. Liu, J. Zou, S. Xu, Y. Wei et al., Electrospun PAN/BaTiO_3_/MXene nanofibrous membrane with significantly improved piezoelectric property for self-powered wearable sensor. Chem. Eng. J. **489**, 151495 (2024). <https://doi.org/10.1016/j.cej.2024.151495>
3. S. Wang, W. Tong, Y. Li, P. Zhang, Y. Liu et al., Contributions of piezoelectricity and triboelectricity to a hydroxyapatite/PVDF–HFP fiber-film nanogenerator. Nano Energy **105**, 108026 (2023). <https://doi.org/10.1016/j.nanoen.2022.108026>
4. J.-X. Chen, J.-W. Li, Z.-J. Jiang, C.-W. Chiu, Polymer-assisted dispersion of reduced graphene oxide in electrospun polyvinylidene fluoride nanofibers for enhanced piezoelectric monitoring of human body movement. Chem. Eng. J. **498**, 155244 (2024). <https://doi.org/10.1016/j.cej.2024.155244>
5. Y. Wen, M. Xu, Y. Hu, J. Bao, Q. Yang et al., Flexible and oriented composite electrospun fiber membrane based on poly(L-lactic acid) for self-powered sensing. Chem. Eng. J. **510**, 161795 (2025). <https://doi.org/10.1016/j.cej.2025.161795>
6. X. Guan, B. Xu, J. Gong, Hierarchically architected polydopamine modified BaTiO_3_@P(VDF-TrFE) nanocomposite fiber mats for flexible piezoelectric nanogenerators and self-powered sensors. Nano Energy **70**, 104516 (2020). <https://doi.org/10.1016/j.nanoen.2020.104516>
7. D. Wang, D. Zhang, P. Li, Z. Yang, Q. Mi et al., Electrospinning of flexible poly(vinyl alcohol)/MXene nanofiber-based humidity sensor self-powered by monolayer molybdenum diselenide piezoelectric nanogenerator. Nano-Micro Lett. **13**(1), 57 (2021). <https://doi.org/10.1007/s40820-020-00580-5>
8. J. Xiong, L. Wang, F. Liang, M. Li, Y. Yabuta et al., Flexible piezoelectric sensor based on two-dimensional topological network of PVDF/DA composite nanofiber membrane. Adv. Fiber Mater. **6**(4), 1212–1228 (2024). <https://doi.org/10.1007/s42765-024-00415-7>
9. T. Liu, D. Qu, L. Guo, G. Zhou, G. Zhang et al., MXene/TPU composite film for humidity sensing and human respiration monitoring. Adv. Sens. Res. **3**(3), 2300014 (2024). <https://doi.org/10.1002/adsr.202300014>
10. L. Ma, R. Wu, A. Patil, S. Zhu, Z. Meng et al., Full-textile wireless flexible humidity sensor for human physiological monitoring. Adv. Funct. Mater. **29**(43), 1904549 (2019). <https://doi.org/10.1002/adfm.201904549>
11. S. Ding, X. Jin, B. Wang, Z. Niu, J. Ma et al., Integrating Ti_3_C_2_T*_x_* MXene nanosheets with thermoplastic polyurethane nanofibers as wearable humidity sensors for noninvasive sleep monitoring and noncontact sensing. ACS Appl. Nano Mater. **6**(13), 11810–11821 (2023). <https://doi.org/10.1021/acsanm.3c01732>
12. D. Lei, Q. Zhang, N. Liu, T. Su, L. Wang et al., Self-powered graphene oxide humidity sensor based on potentiometric humidity transduction mechanism. Adv. Funct. Mater. **32**(10), 2107330 (2022). <https://doi.org/10.1002/adfm.202107330>
13. Q. Zhao, Y. Jiang, Z. Duan, Z. Yuan, J. Zha et al., A Nb2CTx/sodium alginate-based composite film with neuron-like network for self-powered humidity sensing. Chem. Eng. J. **438**, 135588 (2022). <https://doi.org/10.1016/j.cej.2022.135588>
14. S. Li, Y. Zhang, X. Liang, H. Wang, H. Lu et al., Humidity-sensitive chemoelectric flexible sensors based on metal-air redox reaction for health management. Nat. Commun. **13**(1), 5416 (2022). <https://doi.org/10.1038/s41467-022-33133-y>
15. B.-X. Li, Z. Luo, H. Sun, Q. Quan, S. Zhou et al., Spectral-selective and adjustable patterned polydimethylsiloxane/MXene/nanoporous polytetrafluoroethylene metafabric for dynamic infrared camouflage and thermal regulation. Adv. Funct. Mater. **34**(45), 2407644 (2024). <https://doi.org/10.1002/adfm.202407644>
16. J. Mei, H. Liao, S. Yang, M. Liu, H. Tu et al., Multiband electromagnetic interference shielding composite with dynamic thermal management and passive infrared stealth performance. ACS Appl. Mater. Interfaces **17**(30), 43839–43851 (2025). <https://doi.org/10.1021/acsami.5c08956>
17. Y. Bai, B. Zhang, J. Ma, Y. Cheng, P. Cui et al., Adhesion strategy for cross-linking AgNWs/MXene Janus membrane: stretchable and self-healing electromagnetic shielding and infrared stealth capabilities. Small **21**(5), e2408950 (2025). <https://doi.org/10.1002/smll.202408950>
18. Q. Ji, X. Sheng, X. Li, S. Liu, Q. Chen et al., Camel skin-fat structure inspired MXene@PVA/PCC aerogel composite for efficient medium and low temperature infrared stealth. Chem. Eng. J. **476**, 146671 (2023). <https://doi.org/10.1016/j.cej.2023.146671>
19. J. Cui, J. Wu, A. Feng, Y. Yu, L. Mi et al., Low infrared emissivity and oxidation stability of Ti_3_C_2_T_x_ MXene-based composite with tannic acid. Chem. Eng. J. **493**, 152289 (2024). <https://doi.org/10.1016/j.cej.2024.152289>
20. N. Pang, X. Cheng, Y. Wang, X. Yin, X. Meng et al., Flexible multifunctional MXene/SWCNTs composite films with excellent infrared stealth, electromagnetic interference shielding and electrical heating properties. Carbon **238**, 120303 (2025). <https://doi.org/10.1016/j.carbon.2025.120303>
21. K. Sun, F. Meng, X. Zhang, H. Yan, H. Liu et al., Flexible MXene-functionalized composite phase change film with dual-mode of solar-thermal conversion and infrared stealth. Chem. Eng. J. **509**, 161279 (2025). <https://doi.org/10.1016/j.cej.2025.161279>
22. J. Yang, Y. Guo, X. Li, Y. Qiu, X. Wang et al., Fabry-Pérot matching lossy metasurface for coordinated, adaptive, and ultra-broadband visible-infrared-radar compatible camouflage. Adv. Mater. **38**(6), e17422 (2026). <https://doi.org/10.1002/adma.202517422>
23. N. Pang, X. Cheng, X. Yin, Y. Wang, W. Liu et al., Janus-structured ion-bridged MXene@PDA@PNF flexible composite films for synergistic infrared stealth, Joule thermal management, and EMI shielding. J. Mater. Sci. Technol. **259**, 292–306 (2026). <https://doi.org/10.1016/j.jmst.2025.10.007>
